# Sex-related disparities in elderly patients with heart failure with mildly reduced or preserved ejection fraction

**DOI:** 10.1093/eschf/xvaf022

**Published:** 2026-01-08

**Authors:** Emilia D’Elia, Raul Limonta, Cinzia Giaccherini, Christian Basile, Edoardo Sciatti, Ottavio Zucchetti, Luca Di Odoardo, Salvatore D’Isa, Erika Chiesa, Mauro Gori, Lars H Lund, Gianluigi Savarese, Antonello Gavazzi, Michele Senni

**Affiliations:** Cardiovascular Department, Papa Giovanni XXIII Hospital, Piazza OMS, 1, Bergamo 24127, Italy; Department of Medicine and Surgery, Milano Bicocca University, Milano, Italy; Department of Medicine and Surgery, Milano Bicocca University, Milano, Italy; FROM Research Foundation E.T.S., Papa Giovanni XXIII Hospital, Bergamo, Italy; ANMCO Research Center, Heart Care Foundation, Florence, Italy; Department of Clinical Science and Education, Södersjukhuset, Karolinska Institutet, Stockholm, Sweden; Cardiovascular Department, Papa Giovanni XXIII Hospital, Piazza OMS, 1, Bergamo 24127, Italy; Cardiovascular Department, Papa Giovanni XXIII Hospital, Piazza OMS, 1, Bergamo 24127, Italy; Cardiovascular Department, Papa Giovanni XXIII Hospital, Piazza OMS, 1, Bergamo 24127, Italy; Cardiovascular Department, Papa Giovanni XXIII Hospital, Piazza OMS, 1, Bergamo 24127, Italy; FROM Research Foundation E.T.S., Papa Giovanni XXIII Hospital, Bergamo, Italy; Cardiovascular Department, Papa Giovanni XXIII Hospital, Piazza OMS, 1, Bergamo 24127, Italy; Department of Medicine and Surgery, Milano Bicocca University, Milano, Italy; Department of Medicine, Karolinska Institutet, Stockholm, Sweden; Department of Cardiology, Karolinska University Hospital, Stockholm, Sweden; Department of Clinical Science and Education, Södersjukhuset, Karolinska Institutet, Stockholm, Sweden; FROM Research Foundation E.T.S., Papa Giovanni XXIII Hospital, Bergamo, Italy; Cardiovascular Department, Papa Giovanni XXIII Hospital, Piazza OMS, 1, Bergamo 24127, Italy; Department of Medicine and Surgery, Milano Bicocca University, Milano, Italy

**Keywords:** Heart failure, Heart failure with preserved ejection fraction, Heart failure with mildly reduced ejection fraction, Gender disparities, Age disparities

## Abstract

**Introduction:**

Heart failure with preserved or mildly reduced ejection fraction (HFmrEF/HFpEF) is a complex syndrome common in elderly patients with multiple comorbidities. Age and sex affect the clinical phenotypes and outcomes of this condition. This study aimed to identify age- and sex-specific factors influencing prognosis in elderly patients with HFmrEF/HFpEF to improve risk stratification and guide personalized treatment.

**Methods:**

This observational, ambispective study was conducted at Papa Giovanni XXIII Hospital, Bergamo, from June 2017 to August 2022, enrolling patients >65 years with HFmrEF/HFpEF [New York Heart Association (NYHA) Class II–IV] according to ESC guidelines. Data collected included demographics, medical history, echocardiograms, and lab tests. Follow-up lasted at least 1 year, with outcomes defined as a composite of all-cause death, urgent heart transplant, HF hospitalization, and emergency department referral for decompensated HF. Findings were validated using the Swedish HF registry with a similar cohort.

**Results:**

Among 2263 HF patients, 971 HFmrEF/HFpEF patients (56.8% males, mean age 79.2 years) were analysed. Males had a higher prevalence of cardiovascular risk factors (e.g. diabetes, obesity, coronary artery disease). The composite outcome occurred more frequently in males (20.6 vs 17.14 per 100 patient years; IRR = 1.20, *P* = .035). Multivariable analysis identified male sex (HR 1.40, 95% CI 1.13–1.73), age >80 years (HR 1.91, 95% CI 1.22–3.00), higher NYHA class, chronic kidney disease, and severe valvular heart disease as independent predictors of worse outcomes. Males had a 40% higher risk of the outcome compared with women (HR 1.40, 95% CI 1.13–1.73), while patients >80 years old had nearly double the risk compared with those aged 65–70 (HR 1.91, 95% CI 1.22–3.00). The validation analysis in the SwedeHF, adapting the same multiple Cox regression model on 20 950 selected patients, median age 79 years and 57.8% men, and observed between January 2017 and August 2022, showed similar independent risk factors for the composite outcome.

**Conclusion:**

This study highlights significant sex disparities in elderly HFmrEF/HFpEF patients, with higher age and male sex being an independent predictor for poor outcomes. These findings emphasize the need for personalized treatment strategies based on these demographic factors.

## Introduction

Pathophysiological differences in cardiovascular disease between males and females are well-documented, with male sex demonstrating a negative predictive value for morbidity and mortality compared with female sex.^1^ Although females are more frequently affected by heart failure (HF) with mildly reduced and preserved ejection fraction (HFmrEF/HFpEF),^2^ the underlying pathophysiological mechanisms and therapeutic implications of this sex difference remain incompletely understood.

Endothelial dysfunction and neurohormonal activation are central pathophysiological mechanisms in HFmrEF/HFpEF, with hormonal fluctuations influenced by sex and age playing a significant role.^3^ Females often exhibit greater cardiovascular sensitivity to risk factors such as hypertension, diabetes, and obesity, which may contribute to a higher prevalence of HFmrEF/HFpEF.^4^ Hormone replacement therapy in females shows cardiovascular benefits, highlighting oestrogen’s role in cardiac health,^5^ and favouring structural heart differences compared with males.^6^

Despite advances in understanding the pathophysiological mechanisms involved in HFmrEF/HFpEF, the impact of ageing and sex has often been overlooked in clinical studies.^7^ Recent research emphasizes the need to incorporate sex as a prognostic factor, however no prognostic score to date has included sex among its variables.^8^ Furthermore, differences in drug efficacy in the 3-way interaction between age-sex-treatment remain largely underexplored, particularly in elderly populations. Notably, elderly individuals and women are often underrepresented in clinical trials,^9^ and the real-world impact of age- and sex-related comorbidities on outcomes in this subgroup is not well defined. Given the rising prevalence and healthcare burden of long-term survivors with HFmrEF/HFpEF, alongside the persistent underestimation of sex- and age-related factors, identifying prognostic markers for clinical outcomes in the real world is of critical importance from a public health perspective. To address these gaps, we analysed a real-world cohort of elderly patients aged >65 years with a diagnosis of HFmrEF/HFpEF. Additionally, to strengthen the generalizability of our findings, we validated the results using an external cohort derived from the Swedish HF (SwedeHF) registry.

## Methods

### Study design and population

This observational ambispective study was conducted in the Cardiology Department at Papa Giovanni XXIII Hospital, Bergamo, Italy, where cardiologists and internists work closely to enhance the management of HFmrEF/HFpEF by a collaborative approach. Consecutive patients admitted for HF defined according to the European Society of Cardiology (ESC) Guidelines were enrolled from June 2017 to August 2022 into the OPPORTUNITIES registry, aimed at characterizing HF patients both in the acute phase and in the outpatients setting. Follow up of the patients lasted at least 1 year.

### Inclusion and exclusion criteria

Inclusion criteria were an age > 65 years, a diagnosis of HFmrEF/HFpEF and NYHA) Class II–IV. HFpEF was diagnosed based on ESC guideline criteria available at the time of patient inclusion (LVEF >50%, signs/symptoms of HF, and evidence of diastolic dysfunction or structural heart disease). If brain natriuretic peptide (BNP) or (NTproBNP) were not available for the HF diagnosis, a previous HF hospitalization within 1 year was considered as a surrogate for the diagnosis.

Exclusion criteria were an admission for causes other than HF, an acute coronary syndrome in the previous 3 months, and lack of informed consent.

### Data collection

Upon admission, patient demographics, past medical history, medications, echocardiograms, electrocardiograms, and laboratory blood tests were recorded. Echocardiography was performed according to the recommendations of the American Society of Echocardiography,^10,11^ with left ventricular ejection fraction (LVEF) measured using the biplane modified Simpson’s method. Also valvular heart disease (VHD) was evaluated according to international recommendations, and severe VHD was defined according to current ESC and ASE guidelines criteria,^12,13^ based on integrated echocardiographic assessment including valve morphology, jet velocity, vena contracta width, effective regurgitant orifice area, and indexed aortic valve area when applicable. Both degenerative and functional aetiologies were considered.^12,13^ Chronic kidney disease (CKD) was defined as an estimated glomerular filtration rate (eGFR) <60 mL/min/1.73 m^2^, calculated using the CKD-EPI formula. Data on albuminuria or proteinuria were not systematically available and were therefore not included in the definition. Post-discharge follow-up data were collected from medical records or via telephonic interviews.

### Outcomes

The outcome was a composite of all-cause death, urgent heart transplant, hospitalization for heart failure (HHF), and urgent referral to the emergency department (ED) for decompensated HF. ED referrals were identified as HF-related if accompanied by HF signs/symptoms (dyspnoea, oedema), elevated natriuretic peptides, and need for supplementary intravenous diuretic therapy.

### Validation cohort

The findings of this study were validated in the SwedeHF registry, a prospective ongoing quality and research registry of HF patients in Sweden.^14^ This analysis included only patients with a LVEF >40%, age >65 years, and a registration between January 2017 and August 2022. When a patient reported multiple observation, only the latest one was considered. Definition of the variables used in SwedeHF is reported in Supplementary Table S1. Missing values for the variables included in multivariable models were handled with multiple imputation by chained equation (10 datasets generated) with variables marked in Supplementary Table S2. Due to the unavailability of reliable ICD codes for ED visit, the outcome for the validation analysis was a composite of all-cause death, heart transplant, and HF hospitalization.

### Statistical analysis

Patients were stratified based on sex. Continuous variables were reported as medians and interquartile range (IQR), while dichotomous variables were reported as counts and percentages. Differences between groups were evaluated using the Mann–Whitney test for continuous variable and the χ^2^ test (or Fisher’s exact test when appropriate) for dichotomous variables. Univariable and multivariable survival analysis was performed using Cox proportional hazards regression models to assess the association between patient characteristics and the risk of the composite outcome. Variables with a *P*-value <.10 in the univariable analysis were initially included in the multivariable model. A backward stepwise selection method was then applied to identify the final set of covariates retained in the model, based on statistical significance and overall model fit. Hazard ratios (HRs) with 95% confidence intervals (CIs) were estimated to quantify the association between each covariate and the outcomes. Kaplan–Meier curves were fitted to visualize the survival distribution according to sex, and the log-rank test was used to compare survival curves. A *P*-value <.05 was considered significant. Statistical analysis was performed using STATA 16 (StataCorp LLC, College Station, TX, USA).

## Results

The OPPORTUNITIES cohort consists of 2.263 HF patients consecutively enrolled between January 2017 and August 2022. After application of the inclusion and exclusion criteria, 971 patients were included (Supplementary Fig. S1). They were followed for a median observation time of 2.9 years (IQR 1.1–4.7), and for at least 1 year (Table 3).

### Baseline characteristics

Table 1 presents the main characteristics of our study cohort, stratified by sex. Specifically, 56.8% of these patients were male, with a median age of 79.2 years (IQR 73.8–84.0), a median body mass index of 25.3 kg/m^2^ (IQR 22.5–28.3), and NYHA Class II in 62.6%. The majority were enrolled as outpatients (60.1%). The primary HF aetiology was ischaemic heart disease (34.6%), followed by VHD (22.8%) and hypertensive heart disease (13.8%). Only 14.2% of patients had non-ischaemic cardiomyopathy. Hypertension (57%) was the predominant cardiovascular risk factor, followed by diabetes (30%), dyslipidemia (27.6%), and obesity (15.4%). Significant comorbidities included atrial fibrillation (45.4%) and CKD, defined as an estimated glomerular filtration rate (eGFR) <60 mL/min/1.73 m² (37.9%). Other relevant conditions were malignancies (15%), anemia (22.7%), peripheral artery disease (18%), thyroid disorders (14.9%), and respiratory diseases—including asthma and chronic obstructive pulmonary disease (12.8%).

**Table 1:** General characteristics of the population descriptive analysis of the main characteristics of the study cohort stratified by sex

			Sex		
	*N*	Total *N* = 971	M *N* = 552	F *N* = 419	*P*-value
**Demographic**					
** Age (years)**	971	79.2 (73.8–84.0)	78.2 (73.1–83.5)	80.5 (75.1–84.8)	<.001
** Ethnicity**	971				.67
**Caucasian**		958 (99.7)	542 (99.6)	416 (99.8)	
**Hispanic**		2 (0.2)	1 (0.2)	1 (0.2)	
**Other**		1 (0.1)	1 (0.2)	0 (0.0)	
** Diagnoses**	970				<.001
**Ischaemic cardiomyopathy**		336 (34.6)	248 (44.9)	88 (21.1)	
**Non ischaemic cardiomyopathy**		138 (14.2)	82 (14.9)	56 (13.4)	
**Valvular cardiomyopathy**		221 (22.8)	90 (16.3)	131 (31.3)	
**Other cardiomyopathy**		43 (4.4)	32 (5.8)	11 (2.6)	
**Hypertensive cardiomyopathy**		134 (13.8)	63 (11.4)	71 (17.0)	
**Tach cardiomyopathy**		73 (7.5)	24 (4.3)	49 (11.7)	
**Other**		25 (2.6)	13 (2.4)	12 (2.9)	
** Patient setting**	971				.45
**Outpatient**		584 (60.1)	341 (61.8)	243 (58.0)	
**Inpatient (ward)**		349 (35.9)	189 (34.2)	160 (38.2)	
**Day Hospital**		38 (3.9)	22 (4.0)	16 (3.8)	
** NYHA class**	965				.018
**I**		165 (17.1)	108 (19.7)	57 (13.6)	
**IIa**		494 (51.2)	287 (52.5)	207 (49.5)	
**IIb**		110 (11.4)	56 (10.2)	54 (12.9)	
**IIIa**		179 (18.5)	85 (15.5)	94 (22.5)	
**IIIb**		9 (0.9)	6 (1.1)	3 (0.7)	
**IV**		8 (0.8)	5 (0.9)	3 (0.7)	
** Ejection fraction**	971				<.001
**mrEF**		315 (32.4)	217 (39.3)	98 (23.4)	
**pEF**		656 (67.6)	335 (60.7)	321 (76.6)	
** Heart failure type**	971				.14
**De novo**		334 (34.4)	179 (32.4)	155 (37.0)	
**Worsening**		637 (65.6)	373 (67.6)	264 (63.0)	
**Clinical history**					
**MI**	969	190 (19.6)	140 (25.5)	50 (11.9)	<.001
**PTCA**	969	266 (27.5)	191 (34.7)	75 (17.9)	<.001
**Stroke**	969	45 (4.6)	21 (3.8)	24 (5.7)	.16
**Main comorbidities^a^**					
**Atrial fibrillation**	971	441 (45.4)	245 (44.4)	196 (46.8)	.46
**CAD**	971	316 (32.5)	221 (40.0)	95 (22.7)	<.001
**Family history for CAD**	971	53 (5.5)	35 (6.3)	18 (4.3)	.16
**Hypertension**	971	553 (57.0)	313 (56.7)	240 (57.3)	.86
**Diabetes mellitus**	971	299 (30.8)	195 (35.3)	104 (24.8)	<.001
**Hyperuricaemia**	971	96 (9.9)	62 (11.2)	34 (8.1)	.11
**Dyslipidaemia**	971	268 (27.6)	153 (27.7)	115 (27.4)	.93
**Cerebrovascular disease**	971	30 (3.1)	22 (4.0)	8 (1.9)	.064
**Obesity**	971	150 (15.4)	100 (18.1)	50 (11.9)	.008
**Vasculopathy**	971	175 (18.0)	114 (20.7)	61 (14.6)	.014
**Smoking history**	971	54 (5.6)	35 (6.3)	19 (4.5)	.22
**Anaemia**	971	220 (22.7)	119 (21.6)	101 (24.1)	.35
**CKD**	971	368 (37.9)	232 (42.0)	136 (32.5)	.002
**COPD**	971	124 (12.8)	79 (14.3)	45 (10.7)	.099
**Liver disease**	971	53 (5.5)	29 (5.3)	24 (5.7)	.75
**Neoplasia**	971	146 (15.0)	87 (15.8)	59 (14.1)	.47
**Physical examination**					
**Height**	894	167.0 (160.0–172.0)	171.0 (168.0–175.0)	160.0 (155.0–165.0)	<.001
**Weight**	894	70.0 (61.0–80.0)	75.5 (68.0–85.0)	63.2 (54.5–72.0)	<.001
**BMI**	893	25.3 (22.5–28.3)	25.6 (23.4–28.7)	24.5 (21.6–27.6)	<.001
**BMI classes**	893				.002
**<25**		421 (47.1)	212 (42.1)	209 (53.7)	
**25–30**		334 (37.4)	204 (40.5)	130 (33.4)	
**≥30**		138 (15.5)	88 (17.5)	50 (12.9)	
**Systolic BP**	896	122.0 (115.0–140.0)	124.0 (115.0–140.0)	120.0 (110.0–140.0)	.49
**Diastolic BP**	896	70.0 (65.0–80.0)	70.0 (65.0–80.0)	70.0 (64.0–80.0)	.35
**Heart rate**	873	68.0 (60.0–78.0)	67.0 (60.0–76.0)	69.0 (62.0–79.5)	.002
**ECG**					
**Sinus rhythm**	930	494 (53.1)	297 (56.0)	197 (49.3)	.040
**Atrial fibrillation**	931	342 (36.7)	181 (34.2)	161 (40.1)	.060
**LV hypertrophy**	930	105 (11.3)	52 (9.8)	53 (13.3)	.10
**PM**	931	182 (19.5)	105 (19.8)	77 (19.2)	.82
**QRS**	929	108.0 (96.0–126.0)	108.0 (100.0–138.0)	106.0 (90.0–120.0)	<.001
**QT**	810	423.0 (408.0–460.0)	426.0 (408.0–464.0)	418.0 (402.0–454.0)	.018
**QTc**	484	457.0 (432.0–487.0)	457.0 (429.0–491.0)	458.0 (437.0–483.5)	.47
**Heart rate**	837	67.0 (60.0–77.0)	66.0 (60.0–76.0)	68.5 (62.0–80.0)	<.001
**Bundle branch block**	931				.11
**No**		761 (81.7)	424 (80.0)	337 (84.0)	
**RBBB**		81 (8.7)	55 (10.4)	26 (6.5)	
**LBBB**		89 (9.6)	51 (9.6)	38 (9.5)	
**AV block**	931				.010
** 0**		846 (90.9)	469 (88.5)	377 (94.0)	
**1**		81 (8.7)	59 (11.1)	22 (5.5)	
**2**		4 (0.4)	2 (0.4)	2 (0.5)	
**Hemiblock**	931				.48
**0**		828 (88.9)	468 (88.3)	360 (89.8)	
**1**		103 (11.1)	62 (11.7)	41 (10.2)	
**Pharmacological therapy**					
**Beta-blockers**	967	718 (74.3)	398 (72.4)	320 (76.7)	.12
**RASI**	967	597 (61.7)	353 (64.2)	244 (58.5)	.072
**CCB**	967	279 (28.9)	161 (29.3)	118 (28.3)	.74
**MRA**	967	502 (51.9)	281 (51.1)	221 (53.0)	.56
**SGLT-2**	967	8 (0.8)	4 (0.7)	4 (1.0)	.69

Continuous variables are summarized as median and interquartile range, while categorical variables are presented as count and percentage. *P*-value refers to χ^2^ test (or exact Fisher’s test) for categorical variables and to Mann–Whitney *U* test for continuous ones.

NYHA, New York Heart Association; MI, myocardial infarction; PTCA, percutaneous transluminal coronary angioplasty; CAD, coronary artery disease; CKD, chronic kidney disease; COPD, chronic obstructive pulmonary disease; BMI, body mass index; BP, blood pressure; PM, pace-maker; RBBB, right bundle block branch; LBBB, left bundle block branch; RASI, renin angiotensin system inhibitors; CCB, calcium channel blockers.

^a^Active comorbidities documented at the time of enrolment.

Table 2 shows echocardiographic parameters and laboratory data of the population.

**Table 2:** Echocardiographic parameters and laboratory data of the population descriptive analysis of the main characteristics of the study cohort stratified by sex

Echocardiogram	All patients (*n* = 971)	M (*n* = 552)	F (*n* = 419)	*P*-value
**LVEDD (mm)**	50.0 (45.0–55.5)	53.0 (47.0–57.0)	47.0 (42.0–52.0)	<.001
**LVESD (mm)**	37.0 (32.0–43.0)	38.0 (33.0–45.0)	34.0 (30.0–40.0)	<.001
**LVEDV (mL)**	104.5 (80.0–132.5)	123.0 (95.0–149.0)	88.0 (64.0–105.0)	<.001
**LVESV (mL)**	49.0 (32.5–71.5)	59.0 (40.0–80.0)	37.0 (26.0–52.0)	<.001
**LA area (cm^2^)**	26.0 (23.5–31.0)	26.2 (24.0–32.0)	25.9 (22.6–30.0)	.11
**RV diameter (mm)**	41.0 (37.0–46.0)	43.0 (38.0–48.5)	39.0 (35.0–43.0)	.008
**RV TAPSE (mm)**	20.0 (18.0–23.0)	20.0 (18.0–23.0)	20.0 (17.0–23.0)	.64
**PAPs (mmHg)**	35.0 (28.0–45.0)	33.0 (27.0–45.0)	36.0 (29.0–45.0)	.19
**Laboratory**				
**Hb (g/dL)**	12.6 (11.2–13.8)	13.0 (11.3–14.4)	12.2 (11.2–13.3)	<.001
**HTC (%)**	37.3 (33.4–40.7)	37.7 (33.4–41.5)	36.9 (33.4–39.8)	.41
**WBC (×10^9^/L)**	7.0 (6.0–9.4)	7.1 (6.1–9.5)	6.9 (5.9–9.1)	.65
**PLT (×10^9^/L)**	208.0 (178.0–253.0)	198.0 (166.0–238.0)	220.0 (196.0–267.0)	.005
**Ferritin (ng/mL)**	124.5 (45.0–239.0)	150.0 (50.0–347.0)	102.0 (35.0–178.0)	.004
**Blood glucose (mg/dL)**	104.0 (90.0–125.0)	106.0 (91.0–127.0)	100.0 (88.0–120.0)	.090
**HbA1c (%)**	7.3 (6.8–43.0)	7.9 (6.8–43.0)	7.2 (7.0–7.5)	.50
**Serum creatinine (mg/dL)**	1.2 (0.9–1.5)	1.3 (1.0–1.6)	1.1 (0.9–1.5)	<.001
**eGFR (MDRD) (mL/min/1.73)**	52.4 (38.5–68.4)	55.5 (41.7–71.8)	48.1 (34.8–63.2)	<.001
**Blood urea nitrogen (mg/dL)**	71.0 (50.0–104.0)	68.0 (49.0–106.0)	72.0 (51.5–101.5)	.60
**Serum uric acid (mg/dL)**	7.0 (5.7–8.6)	7.0 (5.6–8.6)	6.8 (5.7–8.6)	.87
**Sodium (mmol/L)**	140.0 (138.0–142.0)	140.0 (138.0–142.0)	140.0 (138.0–142.0)	.34
**Potassium (mmol/L)**	4.4 (4.1–4.7)	4.4 (4.1–4.7)	4.4 (4.1–4.6)	.97
**CRP (mg/L)**	0.8 (0.3–1.9)	0.8 (0.3–1.9)	0.8 (0.2–1.8)	.42
**TSH (µIU/mL)**	1.8 (1.0–2.9)	1.6 (0.9–2.7)	1.9 (1.0–3.3)	.11
**Troponin I Hs (ng/L)**	0.2 (0.0–18.5)	0.3 (0.1–17.0)	0.1 (0.0–22.0)	.39
**AST (U/L)**	21.5 (16.0–29.0)	21.0 (17.0–29.0)	22.0 (16.0–29.0)	.94
**ALT (U/L)**	21.0 (15.0–30.0)	22.0 (16.0–32.0)	19.0 (14.0–27.0)	.063
**Total bilirubinaemia (mg/dL)**	0.8 (0.5–1.1)	0.8 (0.5–1.1)	0.8 (0.5–1.0)	.88
**Total cholesterol (mg/dL)**	149.0 (126.0–181.0)	146.0 (123.0–173.5)	155.0 (130.0–191.0)	.005
**HDL (mg/dL)**	44.0 (35.0–53.0)	43.0 (34.0–51.0)	45.0 (37.0–55.0)	.035
**LDL (mg/dL)**	83.0 (63.0–108.6)	81.6 (61.8–102.6)	85.0 (67.2–117.4)	.033
**Triglycerides (mg/dL)**	103.0 (82.0–138.0)	102.0 (79.0–138.5)	104.0 (85.0–138.0)	.36
**BNP (pg/mL)**	243.0 (134.0–447.0)	260 (135–465)	222.0 (129.0–395.0)	.11

LVEDD, left ventricle end diastolic diameter; LVESD, left ventricle end systolic diameter; LVEDV, left ventricle end diastolic volume; LVESV, left ventricle end systolic volume; LA, left atrium; RV, right ventricle; PAPs pulmonary artery pressures; Hb, haemoglobin; HCT, haematocrit; WBC, white blood cell; TSAT, transferrin saturation; eGFR, glomerular filtration rate; CRP, C-reactive protein; TSH, thyroid stimulation hormone; AST, aspartate transferase; ALT, alanine transferase; HDL, high density lipoprotein; LDL, low density protein; BNP, brain natriuretic peptide.

Continuous variables are summarized as median and interquartile range. *P*-value was calculated with Mann–Whitney non-parametric test.

### Sex-based analysis

Our study revealed notable sex-based differences in the general characteristics and in prevalence of cardiovascular risk factors and comorbidities (Table 1). Males had a significantly lower mean age (78.2 vs 80.5 years, *P* < .001), lower NYHA class (*P* < .018) and a significantly higher prevalence of several cardiovascular risk factors, including diabetes (35.3% vs 24.8%, *P* = .001), obesity (18.1% vs 11.9%, *P* = .008), and alcohol consumption (2.1% vs 0.3%, *P* = .04).

They also had a higher prevalence of vascular diseases, including coronary heart disease (40% vs 22.7%, *P* = .001), acute myocardial infarction (25.5% vs 11.9%, *P* = .01), and peripheral artery disease (20.7% vs 14.6%, *P* = .01). Males also had a higher prevalence of CKD (42% vs 32.5%, *P* = .002). Females, on the other hand, had a higher prevalence of depression (5.5% vs 2.4%, *P* = .01), rheumatological disorders (10.3% vs 3.6%, *P* = .001), and thyroid diseases (20% vs 11.1%, *P* = .001). Females also had lower haemoglobin levels (12.2 g/dL vs 13.0 g/dL, *P* = .001), and ferritin levels (102 mcg vs 150 mcg, *P* = .004). On average, males had a higher number of comorbidities compared with females (mean 3.7 ± 1.9 vs 3.1 ± 1.8, *P* < .001), based on the cumulative presence of predefined conditions including hypertension, diabetes, CKD, atrial fibrillation, and anaemia.

Echocardiographically, females had smaller cardiac dimensions, including lower left ventricular end-diastolic diameter (53.0 mm vs 47.0 mm, *P* = .001) and left ventricular end-systolic diameter (38.0 mm vs 34.0 mm, *P* = .001), as well as smaller ventricular sizes and right ventricular diameter. However, no significant differences were found between males and females in right ventricular function, diastolic function, or left atrial volume indexed.

The BNP levels were similar between males and females, with a median of 260.0 (IQR: 135.0–465.0) in males and 222.0 (IQR: 129.0–395.0) in females (*P* = .11).

### Outcome analysis

The clinical composite outcome occurred more frequently in males compared with females (58.2% vs 51.6%, *P* = .04, 20.6 vs 17.1 per 100 patient years; IRR = 1.20, *P* = .035). This difference was driven primarily by death, occurring more frequently in males compared with females (44.9% vs 36.8%, *P* = .010), with similar proportion of patients experiencing HHF (28.4% vs 27.4%, *P* = .73), and ED visits for decompensated HF (7.2% vs 7.4%, for males and females respectively, *P* = .93). Table 3 shows outcomes of the population, with a composite outcome including death, urgent heart transplantation, ED visit for secondary care, and hospitalization for secondary care. Of note, there was no need of urgent heart transplantation for any patients included in the analysis.

**Table 3:** Outcomes of the population

Outcome		M (*n* = 552)	F (*n* = 419)	*P*-value
**Number of HHF**	0.0 (0.0–1.0)	0.0 (0.0–1.0)	0.0 (0.0–1.0)	.64
**At least one HHF**	272 (28.0)	157 (28.4)	115 (27.4)	.73
**Number of HF ED admissions**	0.0 (0.0–0.0)	0.0 (0.0–0.0)	0.0 (0.0–0.0)	.92
**At least one HF ED admission**	71 (7.3)	40 (7.2)	31 (7.4)	.93
**All causes deaths**	402 (41.4)	248 (44.9)	154 (36.8)	.010
**Composite outcome^a^**	537 (55.3)	321 (58.2)	216 (51.6)	.040
**Observation time (years)**	2.9 (1.1–4.7)	2.6 (1.0–4.7)	3.2 (1.2–4.8)	.19

Continuous variables are summarized as median and interquartile range, while categorical variables are presented as count and percentage. *P*-value refers to χ^2^ test (or exact Fisher’s test) for categorical variables and to Mann–Whitney *U* test for continuous ones.

HF, heart failure; HHF, hospitalization for heart failure; ED, emergency department.

^a^The composite outcome includes death, heart transplantation, emergency department visit for secondary care, and hospitalization for secondary care.

Univariable analyses (Table 4) showed that males had a significantly higher risk of the composite outcome (HR 1.19, 95% CI 1.00–1.41). Patients aged 70–80 years and over 80 years had nearly two and three folds higher risk of the composite outcome compared with patients aged 65–70, respectively. Higher NYHA classes were associated with higher risk, with NYHA III–IV presenting a six-fold higher risk. VHD, atrial fibrillation and diabetes were significantly associated with a higher risk by 40%. In contrast, patients on renin-angiotensin system inhibitors and beta-blockers showed a lower risk compared with those not on these medications.

**Table 4:** Univariate survival analysis of the composite outcome using Cox proportional hazards models

	N not missing	HR	95% CI	*P*-value
**Sex**	971			
**F**		ref		
**M**		1.19	1.00–1.41	.048
**Age (years)**	971			
**65–69**		ref		
**70–80**		1.81	1.28–2.56	.001
**>80**		2.85	2.03–4.00	<.001
**BMI**	893			
**<25**		ref		
**25–30**		0.95	0.78–1.15	.591
**>30**		0.93	0.71–1.20	.560
**NYHA class**	965			
**I**		ref		
**II**		2.62	1.94–3.55	<.001
**III–IV**		6.37	4.59–8.84	<.001
**Severe valve disease**	971			
**No**		ref		
**Yes**		1.43	1.19–1.72	<.001
**Atrial fibrillation**	971			
**No**		ref		
**Yes**		1.36	1.14–1.62	.001
**Diabetes mellitus**	971			
**No**		ref		
**Yes**		1.36	1.13–1.63	.001
**Anaemia**	971			
**No**		ref		
**Yes**		1.86	1.49–2.31	<.001
**Hypertension**	971			
**No**		ref		
**Yes**		1.17	0.98–1.38	.082
**Chronic kidney disease**	759			
**No**		ref		
**Yes**		2.59	2.01–3.34	<.001
**RASI**	967			
**No**		ref		
**Yes**		0.54	0.46–0.64	<.001
**Beta-blockers**				
**No**	967	ref		
**Yes**		0.72	0.59–0.86	<.001
**CCB**	967			
**No**		ref		
**Yes**		1.28	1.07–1.54	.008
**Previous MI**	969			
**No**		ref		
**Yes**		1.13	0.92–1.39	.241
**Previous PCI**	969			
**No**		ref		
**Yes**		1.22	1.02–1.47	.030
**Neoplasia**	971			
**No**		ref		
**Yes**		1.24	1.00–1.55	.055
**QRS**	929	1.003	1.000–1.005	.082
**BNP^a^**	411	1.008	1.001–1.015	.027

Hazard ratios (HR) and corresponding 95% confidence intervals (CI) are presented.

BMI, body mass index; NYHA, New York Heart Association; RASI, renin aldosterone system inhibitors; CCB, calcium channel blockers; MI, myocardial infarction; PCI, percutaneous coronary intervention; BNP, brain natriuretic peptide.

^a^HR and CI provided for increments of 50 units of BNP. Only 411 observations available.

In the final multivariable model (Table 5), male sex (HR 1.40, 95% CI 1.13–1.73), older age (age > 80 years vs age 65–70 years: HR 1.91, 95% CI 1.22–3.00), higher NYHA classes (II vs I HR 2.32, 95% CI 1.52–3.54; III/IV vs I HR 5.67, 95% CI 3.61–8.91), severe VHD (HR 1.39, 95% CI 1.11–1.74), and CKD (HR 1.84, 95% CI 1.38–2.44) were independently associated with a higher risk of the composite outcome.

**Table 5:** Multivariate Cox proportional hazards models for the composite outcome

	HR	95% CI	*P*-value
**Sex**			
**F**	ref		
**M**	1.40	1.13–1.73	.002
**Age (years)**			
**60–69**	ref		
**70–80**	1.48	0.94–2.32	.092
**>80**	1.91	1.22–3.00	.005
**NYHA class**			
**I**	ref		
**II**	2.32	1.52–3.54	<.001
**III–IV**	5.67	3.61–8.91	<.001
**Severe valve disease**			
**No**	ref		
**Yes**	1.39	1.11–1.74	.004
**Anaemia**			
**No**	ref		
**Yes**	1.25	0.98–1.59	.072
**Chronic kidney disease**			
**No**	ref		
**Yes**	1.84	1.38–2.44	<.001
**Neoplasia**			
**No**	ref		
**Yes**	1.27	0.98–1.66	.072

Hazard ratios (HR) and corresponding 95% confidence intervals (CI) are presented.

**Figure 1:**
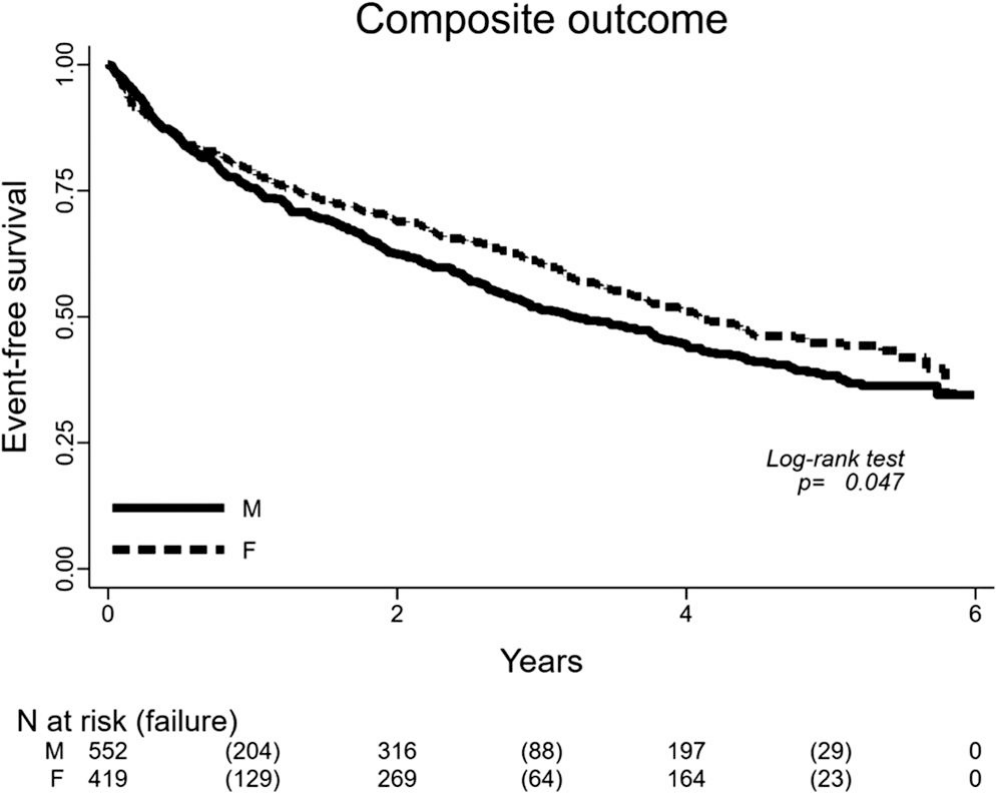
Kaplan–Meier curves according to gender for all-cause mortality, urgent heart transplantation, HHF, and emergency department visits for HF

Figure 1 reports the Kaplan–Meier curves according to gender for the composite outcome, namely all-cause mortality, heart transplantation, HHF, and ED visits for HF. Male sex had a lower outcome-free survival (*P* = .047), with curves separating as early as before 1 year of follow-up.

### Validation with Swedish Heart Registry

Baseline characteristics of the selected patients from SwedeHF are shown in Supplementary Table S2. After applying the inclusion and exclusion criteria, 20 950 patients (median age 79 years [IQR 74–84], 42.2% females) followed for a median of 2.3 years (IQR 1.3–4.0) were included.

Forty-nine per cent of patients experienced the composite outcome, with similar incidence between males (48.9%) and females (49.3%). At the multivariable Cox regression model fitted with the same variables as in the main analysis, male sex (HR 1.12, 95% CI 1.07–1.17), older age (age 70–80 vs 65–69 years HR 1.32, 95% CI 1.21–1.44; age > 80 years vs 65–69 years HR 2.07, 95% CI 1.90–2.26), higher NYHA classes (II vs I HR 1.46, 95% CI 1.33–1.60; III/IV vs I HR 2.64, 95% CI 2.42–2.88), severe VHD (HR 1.25, 95% CI 1.20–1.30), and CKD (HR 1.48, 95% CI 1.42–1.55) were independently associated with a higher risk for the composite outcome (Supplementary Table S3), which was consistent with the main analysis. Furthermore, anaemia (HR 1.58, 95% CI 1.51–1.65) and previous cancer (HR 1.23, 95% CI 1.17–1.30) were also independently associated with higher risk of the composite outcome.

## Discussion

This study highlights significant sex-related differences in cardiovascular risk factors and outcomes among patients aged ≥65 years with HFmrEF/HFpEF. Specifically, we identified five key variables (male sex, older age, higher NYHA class, severe VHD, CKD) that were independently associated with the composite outcome in both the OPPORTUNITIES and SwedeHF registries. While HFmrEF/HFpEF is generally more prevalent among females in population-based studies,^15–17^ the male predominance observed in our real-world hospitalized OPPORTUNITIES cohort from a tertiary care centre may be explained by a higher burden of comorbidities and clinical severity leading to hospitalization in men. In this cohort of 971 patients, in fact, males had a greater prevalence of traditional cardiovascular risk factors, including diabetes, obesity, coronary artery disease, as well as a history of acute myocardial infarction and prior percutaneous coronary interventions.

Of note, these gender prevalences appear to be similar to the data reported by the validation cohort obtained from SwedeHF registry (43.2% females and 56.8% males in the OPPORTUNITIES cohort vs 42.2% and 57.8% in the SwedeHF cohort).

Older age, more advanced NYHA classes, severe VHD, and CKD were independent predictors of adverse clinical outcomes. Notably, patients over 80 years old had nearly double risk of adverse events compared with those aged 65–69. These findings underscore the importance of considering not only sex and age but also comorbid conditions when managing and assessing the prognosis of HFmrEF/HFpEF patients.

Given the complex nature of HFmrEF/HFpEF, an integrated care approach is essential. Traditionally managed by general practitioners or within internal medicine wards, the involvement of both cardiologists and internists is crucial for addressing the multifaceted aspects of the syndrome.^18–20^ In Bergamo, the Cardiovascular Department of the Papa Giovanni XXIII Hospital has successfully established a multidisciplinary approach that ensures comprehensive care targeting both cardiac and systemic health, with the goal of improving patient outcomes.^21^

The Framingham Heart Study and the Rotterdam Study showed that the lifetime risk of HF is similar across sexes, with males at a 21% risk and females at 20% by age 40, and at age 55, 33% for males and 29% for females.^16,17^ However, more recent epidemiological data from Olmsted County reveal a growing disparity. Among 2762 incident HF cases between 2000 and 2010, the proportion of HFpEF cases increased from 48% in 2000–2003 to 52% in 2008–2010, with females nearly twice as likely as males to develop HFpEF. This highlights the increasing burden of HFpEF, which is heavily influenced by comorbidities, unlike HFrEF, where cardiovascular events play a more direct role in mortality. In HFpEF, the progression of comorbidities often drives mortality, underscoring the importance of therapies that target the broader cardio-renal and metabolic continuum.

Medications such as SGLT2 inhibitors, GLP1 receptor agonists, and mineralocorticoid receptor antagonists like spironolactone or finerenone, have demonstrated benefit in this regard, addressing not only HF but also the systemic conditions that contribute to its pathophysiology.^9,22,23^ Our study population showed a high prevalence of arterial hypertension, diabetes, dyslipidaemia, and obesity—factors that contribute to the inflammatory environment underlying HFpEF. Systemic inflammation has thus emerged as a promising target for therapeutic interventions, with clinical trials such as the HERMES trial (ziltivekimab) and the COLT-HF trial (colchicine) exploring this avenue.^24,25^

In our cohort, males had a higher prevalence of diabetes, obesity, and CKD compared with females, though there were no significant sex differences for neoplasms and anaemia.

Given the result of the multivariable Cox proportional hazard model, we focused on a more detailed description of the individual predictors below.

### Age, sex, and New York Heart Association functional class

Several studies have highlighted age as a critical risk factor influencing long-term prognosis. Tromp et al. observed that younger patients with HFpEF, predominantly males with obesity, had higher rates of cardiovascular-related deaths, particularly sudden cardiac death, compared with older patients, who had a higher prevalence of other comorbidities and were more likely to die from non-cardiovascular causes.^26^ Consistently, our study, which included a cohort of patients with a median age of 79.2 years and a significant burden of comorbidities, reaffirmed age as an independent risk factor in this population; notably, we observed a progressively higher risk with each decade of age, from 65 years onward. Although female sex is notably more prevalent among HFpEF patients regardless of age, in our cohort males constituted the predominant group (56.8%). The precise pathophysiological mechanisms underlying sex differences in HFpEF aetiology remain unclear.^27^ Several mechanisms may contribute to the observed sex-related differences in clinical outcomes among elderly patients with HFmrEF/HFpEF. Hormonal influences, particularly oestrogen-related cardioprotective effects, have been shown to modulate vascular tone, endothelial function, and myocardial remodelling, and may partly explain the more favourable prognosis observed in females. In addition, sex-based differences in systemic inflammatory responses and fibrotic pathways have been proposed, with women generally exhibiting higher levels of pro-inflammatory markers but also more preserved microvascular function. Behavioural and psychosocial factors may also play a role: women are often more likely to report symptoms, seek medical attention earlier, and demonstrate greater adherence to therapy, while men may underreport early signs of decompensation. Together, these biological and behavioural differences may underlie the sex-based disparities in disease trajectory and outcomes, and warrant further mechanistic and interventional investigation.

Moreover, in our study, male sex was associated with a higher risk of the composite outcome, and this aligns with robust data supporting that female patients with HFpEF generally have more favourable outcomes than their male counterparts, including lower rates of in-hospital mortality. However, a retrospective analysis of the TOPCAT trial did not find a notable difference in cardiovascular and all-cause mortality between sexes, although females did report poorer patient-reported outcomes and mean age was lower with respect to our data.^28^ Specifically, our results showed that male patients exhibited a 40% higher risk compared with females, suggesting that the female sex may confer a potential protective effect.

This result may be due to several factors, such as the higher cumulative burden of cardiovascular, metabolic and renal comorbidities in males, which, *per se*, worsen prognosis. As far as NHYA class is concerned, higher NYHA classes were associated with higher risk, with NYHA III–IV presenting a six-fold higher risk.

### Chronic kidney disease

In our cohort, 37.9% of patients with HFmrEF/HFpEF had coexisting CKD, with a higher prevalence in males than in females. This is consistent with data showing that CKD is present in 26%–49% of HFpEF patients in large observational studies.^29–32^ Notably, CKD was associated with an approximately 84% increased risk of experiencing the composite adverse outcome. These findings align with broader research, including studies like SwedeHF, which reported a higher prevalence of CKD in HFpEF (56%) compared with HFmrEF (48%) and HFrEF (45%). The overlapping treatment needs of HFpEF and CKD are supported by several key trials, such as the DAPA-CKD and EMPA-KIDNEY trials,^33,34^ which demonstrated the benefits of SGLT2 inhibitors for patients with both CKD and HF, and the FIDELIO-DKD and FIGARO-DKD trials,^35,36^ which showed that finerenone reduced cardiovascular events and slowed disease progression in CKD patients with diabetes.

Interestingly, elevated blood levels of aldosterone and noradrenaline, commonly seen in patients with CKD, downregulate the expression of transferrin receptor 1 (TfR1) induced by iron deficiency, leading to a decreased uptake of iron by cardiomyocytes. Given this evidence, it has been recently hypothesized that this mechanism might have the potential to translate into lack of clinical improvement and worse outcomes from monotherapy with iron supplementation, whereas co-treatment with SGLT2 inhibitors or aldosterone receptor antagonists could yield better outcomes in patients with both HF and CKD.^37,38^ It has also recently been demonstrated that a potential interaction between the mechanism of action of mineralocorticoid receptor antagonists and iron metabolism could be present in this setting.^39^

### Valvular heart disease

Valvular heart disease (VHD) represents a specific clinical entity with a potentially distinct course from other forms of HFpEF.^40,41^ Severe aortic or mitral disease often leads to HF symptoms that may improve after surgical or percutaneous correction.^42^ In our cohort, patients with severe VHD were included only when HF was the dominant clinical presentation, and VHD was therefore considered a secondary contributor rather than the primary diagnosis. Patients with a clear indication for surgical or percutaneous valve intervention, including those excluded from intervention because of prohibitive risk, were not considered in our study population. This approach aligns with other large registries, such as SwedeHF, where VHD is included as a comorbidity rather than an exclusion criterion, to better capture real-world complexity. Nevertheless, we acknowledge that residual confounding by unmeasured valve disease severity or timing of intervention cannot be excluded, and this has been highlighted as a study limitation.

### Comparison with the SwedeHF registry and previous literature

The findings of our real-world cohort were validated against the SwedeHF registry, a large nationwide prospective database with a comparable age and sex distribution. Despite differences in healthcare settings and patient case mix, both cohorts consistently identified older age, male sex, advanced NYHA class, severe valvular heart disease, and CKD as independent predictors of adverse outcomes. This strong reproducibility across distinct populations supports the generalizability of our results. Notably, the SwedeHF registry has long served as a reliable tool for ongoing quality monitoring and clinical research in HF, providing a robust foundation for external comparisons.^14^

Our findings are also in line with previous studies investigating sex-related differences in HFpEF and HFmrEF. In a large outpatient cohort, Russo et at demonstrated that women were more frequently affected by HFpEF, were older, and had fewer ischaemic risk factors, but more non-cardiac comorbidities such as renal dysfunction and anaemia. While overall outcomes appeared similar between sexes, adjusted analyses showed lower mortality in women with HFpEF and HFmrEF. These results highlight the prognostic impact of sex and comorbidities across the ejection fraction spectrum.^43^ Data from the SwedeHF registry documented that over 47.000 patients, reduced kidney function emerged as a strong and independent predictor of mortality, with risk increasing progressively as eGFR declined, even after adjustment for age, NYHA class, HF duration, and comorbidities.^44^ Similarly, analyses involving over 100.000 patients confirmed that individuals with HFpEF were typically older, more often female, and presented a higher prevalence of hypertension, atrial fibrillation, VHD, and anaemia compared with those with HFrEF or HFmrEF.^45^

Importantly, our study expands upon the existing literature by specifically focusing on a high-risk elderly population and by incorporating ED visits into the composite outcome, elements that are rarely included in similar studies. This broader endpoint provides a more comprehensive representation of disease burden in real-world clinical practice and may inform more personalized management strategies for this growing patient population.

### Study limitations

Several limitations should be acknowledged in our study.

First, this is an observational ambispective study, which may introduce selection bias and limit the generalizability of the findings. However, in our department, most patients are admitted with a primary diagnosis of HF, whereas in other hospital units, HF is more often recorded as a secondary diagnosis.

Second, although we are aware of the relevance of assessing diastolic function parameters, including *E* velocity, *eʹ*, and *E*/*e*ʹ, in our dataset these specific echocardiographic values were missing in a substantial proportion of patients, thereby limiting the reliability and interpretability. For this reason, we have not included them in the analysis. Also, we acknowledge that the HFA-PEFF scoring system was not systematically applied for the diagnosis of HFpEF.

Third, detailed data on the use of specific HFmrEF/HFpEF therapies and adherence were not available, potentially confounding the associations with the outcome. Moreover, some specific drug as SGLT2i were still not recommended for HF management at the time of Opportunities Registry was conducted, and the majority of the patients were off this therapy.

Fourth, in our study, we included patients with severe valvular heart disease (VHD), but considered it a secondary condition in HFpEF, not a primary diagnosis. Severe VHD, such as aortic stenosis and mitral regurgitation, is often associated with HFpEF and can worsen prognosis, but was not treated as a primary cause in our study.

Fifth, there was a lack of consistent information on cause-specific mortality. Although all-cause death was systematically recorded, detailed classification into cardiovascular versus non-cardiovascular causes was not reliably available from clinical records, particularly in patients who died outside the hospital or were managed in non-cardiology settings. As a result, we were unable to perform cause-specific survival analyses, which may have provided additional mechanistic insight. This limitation is common in real-world observational studies and reflects the complexity of outcome attribution in elderly multimorbid populations. Another relevant consideration in elderly HFpEF populations is the potential presence of infiltrative or hypertrophic cardiomyopathies, particularly transthyretin amyloidosis (ATTRwt), which is significantly more prevalent among elderly men. Although only a small proportion (<2%) of patients in our cohort had a confirmed diagnosis, this likely underestimates the true prevalence due to limited systematic screening during the study period. ATTRwt may therefore represent an under-recognized contributor to the observed sex disparities in prognosis.

Lastly, we did not assess trace element concentrations, which may accumulate with age and contribute to myocardial dysfunction. This aspect deserves investigation in future studies exploring the pathophysiological links between age, HFmrEF/HFpEF, and systemic toxicity.

## Conclusion

In this large real-world cohort of elderly HFmrEF/HFpEF patients, our study revealed notable differences in clinical characteristics and outcomes based on sex and age, emphasizing the importance of considering these factors in patient management. Future research should focus on further elucidating the mechanisms behind these disparities and developing personalized treatment strategies to improve outcomes for this diverse patient population.

## Supplementary Material

xvaf022_Supplementary_Data

## Declarations

### Data Availability

No data were generated or analysed for this manuscript.

### Pre-registered Clinical Trial Number

None supplied.
