# Supplementary material for: Sex-related disparities in elderly patients with heart failure with mildly reduced or preserved ejection fraction
Source: ESC Heart Fail. 2026 Jan 8;13(1):xvaf022. doi: 10.1093/eschf/xvaf022 (PMC13108282; doi:10.1093/eschf/xvaf022)
Supplement: xvaf022_Supplementary_Data [file xvaf022_supplementary_data.zip › Supplementary_Material_Bergamo.docx]

# **Supplementary Table 1.** Variable definitions and sources

| **Comorbidity** | **Definition** | **Source** | **Time period** |
| --- | --- | --- | --- |
| Obesity | Body mass index ≥30Kg/m2 | SwedeHF | Baseline registration |
| Hypertension | Diagnosis in SwedeHF or in National Patient Register (ICD: I10-I15) | SwedeHF and National Patient Register | Within 5 years |
| Diabetes | Diagnosis in SwedeHF or in National Patient Register (ICD: E10-E14) | SwedeHF and National Patient Register | Within 5 years |
| Ischemic heart disease | Diagnosis in SwedeHF or in National Patient Register (ICD: 410-414, I20-I25; procedure codes: FNG, FNA, FNB, FNC, FND, FNE, FNF, FNH, Z951, Z955). | SwedeHF and National Patient Register | No time restriction |
| Previous acute coronary syndrome | ICD codes 410, 412, I21, I22, I252 | National Patient Register | No time restriction |
| Previous percutaneous coronary intervention | OP code FNG | National Patient Register | No time restriction |
| Atrial fibrillation | Diagnosis in SwedeHF (history of atrial fibrillation or ECG showing atrial fibrillation) or in NPR (ICD: I48). | SwedeHF and National Patient Register | Within 5 years |
| Valvular heart disease | Diagnosis in SwedeHF or in National Patient Register (ICD: A520, I05-I08, I091, I098, I34-I39, Q230-Q233, Z952, Z954) | SwedeHF and National Patient Register | Within 5 years |
| Chronic kidney disease | Estimated glomerular filtration rate (eGFR) <60 mL/min/1.73 m^2^ (CKD-EPI 2021) | SwedeHF | Baseline registration |
| Anemia | Haemoglobin <13 g/L (8.1 mmol/L) in men and <12 g/L (7.5 mmol/L) in non-pregnant women | SwedeHF | Baseline registration |
| Chronic obstructive pulmonary disease | ICD: J40-J44 | National Patient Register | Within 5 years |
| Peripheral artery disease | ICD: I70-I73 | National Patient Register | Within 5 years |
| Stroke | ICD: 430-434, 438, I60-I64, I690-I694 | National Patient Register | No time restriction |
| Dementia | ICD: F00-F03 | National Patient Register | Within 5 years |
| Cancer within the last 3 years | ICD: C00-C26, C30-C34, C37-C41, C43, C45-C58, C60-C76, C81-C85, C88, C90-C97 | National Patient Register | Within 3 years |
| Liver disease | ICD codes B18, I85, I864, I982, K70, K710, K711, K713-7, K72-4, K760, K762-9 | National Patient Register | Within 5 years |
| Depression | ICD codes F32-4 | National Patient Register | Within 5 years |
| Alcohol use disorder | ICD codes E244, E52, F10, G312, G621, G721, I426, K292, K70, K860, O354, P043, Q860, T51, Z502, Z714 ed Ekod: Y90, Y91 | National Patient Register | Within 5 years |
| **Outcomes** | | | |
| Hospitalization for heart failure | Main diagnosis in National Patient Register (ICD: I50, I42, I43, I255, K761, I110, I130, I132, J81) | National Patient Register |  |
| Heart transplant | Main diagnosis in National Patient Register (ICD: FQA00, FQA10, FQA20, FQA30, FQA40, FQA96, Z94.1 ) | National Patient Register |  |

# **Supplementary Table 2.** Baseline characteristics of the enrolled SwedeHF population together with percentage of missing values

| **Variable** | Overall  N=20,950 | Male  N=12,113 (57.8%) | Female  N=8837 (42.2%) | p-value (male vs female) | % Missing |
| --- | --- | --- | --- | --- | --- |
| **Demographics/organizational** | | | | | |
| Age* (years), median [IQR] | 79.0 (74.0-84.0) | 78.0 (73.0-83.0) | 80.0 (75.0-85.0) | <0.001 | 0 |
| Location* (%) |  |  |  | <0.001 | 0 |
| Outpatient | 81 | 76.4 | 84.3 |  |  |
| Inpatient | 19 | 23.6 | 15.7 |  |  |
| **Heart failure characteristics (%)** | | | | | |
| Etiology* |  |  |  | <0.001 | 26.3 |
| Ischemic | 34.1 | 40.7 | 24.8 |  |  |
| Valvular | 8.8 | 8.6 | 9.2 |  |  |
| Hypertensive | 31.1 | 26.3 | 38.0 |  |  |
| Other | 26 | 24.4 | 28 |  |  |
| NYHA class* |  |  |  | <0.001 | 27.3 |
| I | 12.7 | 14.8 | 9.6 |  |  |
| II | 50.4 | 50.8 | 49.9 |  |  |
| III | 35.7 | 33.3 | 39.0 |  |  |
| IV | 1.3 | 1.1 | 1.5 |  |  |
| **Comorbidities (%)** | | | | | |
| Atrial fibrillation* | 66.7 | 68.5 | 64.2 | <0.001 | 0 |
| Ischemic heart disease* | 49.1 | 54.7 | 41.5 | <0.001 | 0 |
| Previous acute coronary syndrome* | 32.6 | 37.1 | 26.4 | <0.001 | 0 |
| Previous percutaneous coronary intervention* | 21.7 | 26.5 | 15.1 | <0.001 | 0 |
| Arterial hypertension* | 80.1 | 78.9 | 81.8 | <0.001 | 0 |
| Previous stroke* | 14.5 | 15.0 | 13.8 | 0.013 | 0 |
| Diabetes* | 28.0 | 30.5 | 24.5 | <0.001 | 0 |
| Obesity* | 27.7 | 25.8 | 30.4 | <0.001 | 27.4 |
| Peripheral artery disease* | 9.9 | 11.3 | 7.9 | <0.001 | 0 |
| Active smoker* | 6.5 | 6.7 | 6.1 | 0.17 | 32.2 |
| Anemia* | 37.8 | 42.4 | 31.6 | <0.001 | 12.9 |
| Chronic Kidney disease* (eGFR < 60 mL/min/m^2^) | 45.9 | 41.4 | 52.0 | <0.001 | 4.8 |
| Chronic obstructive pulmonary disease* | 14.6 | 14.0 | 15.4 | 0.004 | 0 |
| Liver disease* | 2.0 | 2.1 | 1.8 | 0.19 | 0 |
| Dementia* | 1.4 | 1.3 | 1.6 | 0.18 | 0 |
| Depression* | 2.5 | 1.9 | 3.2 | <0.001 | 0 |
| Alcohol use disorder* | 2.3 | 3.3 | 1.0 | <0.001 | 38.3 |
| Cancer in the previous 3 years* | 15.8 | 18.1 | 12.5 | <0.001 | 0 |
| Valvular disease* | 29.2 | 29.0 | 29.5 | 0.46 | 0 |
| **Clinical characteristics** | | | | | |
| Height (cm), median [IQR] | 171.0 (163.0-178.0) | 177.0 (172.0-182.0) | 163.0 (158.0-167.0) | <0.001 | 17.2 |
| Weight (Kg), median [IQR] | 79.0 (68.0-91.0) | 84.0 (75.0-95.0) | 70.4 (60.7-82.7) | <0.001 | 16.6 |
| BMI* (Kg/m^2^), median [IQR] | 26.8 (23.8-30.5) | 26.8 (24.1-30.2) | 26.8 (23.1-31.2) | 0.02 | 27.4 |
| BMI classes |  |  |  | <0.001 | 27.4 |
| <25 | 34.8 | 32.9 | 37.4 |  |  |
| 25-30 | 37.5 | 41.3 | 32.2 |  |  |
| ≥30 | 27.7 | 25.8 | 30.4 |  |  |
| Systolic blood pressure* (mmHg), median [IQR] | 130.0 (116.0-141.0) | 129.0 (115.0-140.0) | 130.0 (119.0-145.0) | <0.001 | 5.3 |
| Diastolic blood pressure* (mmHg), median [IQR] | 72.0 (65.0-80.0) | 72.0 (65.0-80.0) | 72.0 (65.0-80.0) | 0.38 | 5.2 |
| Heart rate* (bpm), median [IQR] | 70.0 (62.0-80.0) | 69.0 (60.0-78.0) | 72.0 (64.0-82.0) | <0.001 | 6.5 |
| **Electrocardiogram at the visit** | | | | | |
| Rythm* (%) |  |  |  | <0.001 | 11.6 |
| Sinus rhythm | 45.4 | 43.7 | 47.7 |  |  |
| Atrial fibrillation | 42.1 | 41.7 | 42.6 |  |  |
| Pacemaker use | 12.5 | 14.6 | 9.7 |  |  |
| QRS* (ms), median [IQR] | 102.0 (90.0-130.0) | 107.0 (94.0-138.0) | 94.0 (84.0-118.0) | <0.001 | 16.4 |
| Left bundle branch block* (%) | 12.5 | 13.0 | 11.9 | 0.03 | 24.9 |
| **Laboratory** | | | | | |
| Hemoglobin (g/dL), median [IQR] | 13.0 (11.8-14.2) | 13.3 (12.0-14.5) | 12.7 (11.6-13.7) | <0.001 | 12.9 |
| Ferritin (μg/L), median [IQR] | 135.0 (65.0-250.7) | 151.0 (76.0-273.0) | 109.0 (51.0-215.0) | <0.001 | 70.4 |
| Transferrin saturation (%), median [IQR] | 23.0 (16.0-32.0) | 25.0 (17.0-33.0) | 22.0 (14.0-30.0) | <0.001 | 75.4 |
| Creatinin (mg/dL), median [IQR] | 1.1 (0.9-1.4) | 1.1 (0.9-1.4) | 1.0 (0.8-1.2) | <0.001 | 4.9 |
| eGFR* (mL/min/1.93 m^2^), median [IQR] | 62.5 (47.0-79.4) | 65.3 (49.4-82.2) | 58.7 (44.1-74.8) | <0.001 | 4.8 |
| Sodium (mEq/L), median [IQR] | 140.0 (138.0-142.0) | 140.0 (138.0-142.0) | 140.0 (138.0-142.0) | 0.02 | 8.2 |
| Potassium* (mEq/L), median [IQR] | 4.2 (3.9-4.5) | 4.2 (4.0-4.5) | 4.2 (3.9-4.5) | <0.001 | 5.6 |
| NT-proBNP* (pg/L), median [IQR] | 1700.0 (758.0-3438.2) | 1611.5 (711.0-3300.0) | 1811.0 (821.7-3643.2) | <0.001 | 19.7 |
| **Treatments (%)** |  |  |  |  |  |
| RASi* | 80.1 | 82.4 | 77.0 | <0.001 | 0.2 |
| Beta-blockers* | 86.4 | 85.5 | 87.6 | <0.001 | 0.1 |

* Variables included in the multiple imputation model together with the index year and the clinical outcome as Nelson-Aelen estimator. BMI, body mass index; eGFR, estimate glomerular filtration rate; IQR, interquartile range; NT-proBNP, N-terminal prohormone of brain natriuretic peptide; NYHA, New York Heart Assoiciation; RASi, renin-angiotenisin system inhibitors

**Supplementary Table 3. Uni- and Multi-variable Cox regression models in the Swedish Heart Failure Registry for the composite clinical outcome**

| **Variabiles** | **Univariable** | | | **Multivariable** | | |
| --- | --- | --- | --- | --- | --- | --- |
|  | **HR** | **CI 95%** | **p-value** | **HR** | **CI 95%** | **p-value** |
| **Sex** |  |  |  |  |  |  |
| Female | ref |  |  | ref |  |  |
| Male | 1.19 | 1.00-1.41 | 0.048 | 1.12 | 1.07-1.17 | <0.001 |
| **Age (years)**  65-69  70-80  >80 | ref  1.81  2.85 | 1.28-2.56  2.03-4.00 | 0.001  <0.001 | ref  1.32  2.07 | 1.21-1.44  1.90-2.26 | <0.001  <0.001 |
| **NYHA class**  I  II  III-IV | ref  2.62  6.37 | 1.94-3.55  4.59-8.84 | <0.001  <0.001 | ref  1.46  2.64 | 1.33-1.60  2.42-2.88 | <0.001  <0.001 |
| **Severe Valve**  No  Yes | ref  1.43 | 1.19-1.72 | <0.001 | ref  1.25 | 1.20-1.30 | <0.001 |
| **CKD**  No  Yes | ref  2.59 | 2.01-3.34 | <0.001 | ref  1.48 | 1.42-1.55 | <0.001 |
| **Anemia**  No  Yes | ref  1.86 | 1.49-2.31 | <0.001 | ref  1.58 | 1.51-1.65 | <0.001 |
| **Previous cancer**  No  Yes | ref  1.24 | 1.00-1.55 | 0.055 | ref  1.23 | 1.17-1.30 | <0.001 |

CI, confidence interval; CKD, chronic kidney disease; HR, hazard ratio; NYHA, New York Heart Association.
