# Supplementary figures and images for: Sex-related disparities in elderly patients with heart failure with mildly reduced or preserved ejection fraction
Source: ESC Heart Fail. 2026 Jan 8;13(1):xvaf022. doi: 10.1093/eschf/xvaf022 (PMC13108282; doi:10.1093/eschf/xvaf022)

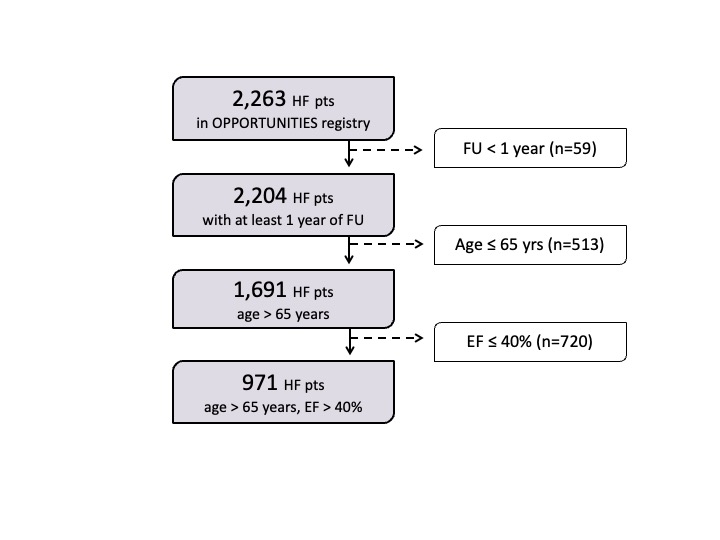

Supplement: xvaf022_Supplementary_Data [file xvaf022_supplementary_data.zip › SupplFig1.jpeg]
